# Perceived Pathways of Change in an Interpersonal Violence Intervention for Mothers: The Importance of Self-Compassion

**DOI:** 10.3390/bs15060739

**Published:** 2025-05-27

**Authors:** Kirsten MacAulay, Naomi C. Z. Andrews, Mary Motz, Gloria J. Lee, Debra J. Pepler

**Affiliations:** 1 Department of Child and Youth Studies, Brock University, 1812 Sir Isaac Brock Way, St. Catharines, ON L2S 3A1, Canada; km20yj@brocku.ca; 2 Early Intervention Department, Mothercraft, 393 King Street East, Toronto, ON M5A 1L3, Canada; mary.motz@mothercraft.org (M.M.); gloria.lee@mothercraft.org (G.J.L.); 3 Department of Psychology, York University, 4700 Keele Street, Toronto, ON M3J 1P3, Canada; pepler@yorku.ca

**Keywords:** intimate partner violence, violence against women, intervention, parenting self-compassion

## Abstract

Interventions to support mothers experiencing interpersonal violence are critical, yet most evaluation research focuses on outcome evaluation, rather than understanding the pathways of change. The goal of the current study was to understand, via mothers’ own perspectives, the specific key pathways through which participation in an interpersonal violence intervention resulted in perceptions of change for mothers and their children. The participants (*N* = 43, 18–43 years old) were mothers who attended and completed a 6–8-week interpersonal violence intervention within 11 community organizations across Canada. Approximately 1–2 months following the intervention, participants completed semi-structured interviews or focus groups wherein they were asked open-ended questions about their experiences in the intervention. Using a phenomenological approach, the results indicated that (1) mothers were able to identify changes in their cognitions and behaviors across three key domains (self, relationships, and parenting), (2) mothers linked these changes to their experiences in the intervention. We integrated and mapped the perceived pathways of change experienced by mothers, which included critical pathways involving self-compassion and self-forgiveness leading to self-esteem and empowerment. The results have implications for our ability to effectively deliver this interpersonal violence intervention, as well as more broadly to improve our understanding of the pathways of change for mothers experiencing violence in relationships.

## 1. Introduction

Interpersonal violence (IPV) is a global public health issue, impacting close to one in three women worldwide (World Health Organization [WHO], 2024), with the potential to significantly impact not only an individual’s life, but also the family and greater society (Renner et al., 2020; Vass & Haj-Yahia, 2023). IPV can lead to extensive negative ramifications that encompass all areas of a woman’s health and well-being, ranging from short-term effects to lifelong chronic conditions (Craven et al., 2023; Graham-Bermann & Miller-Graff, 2015). Often referred to as intimate violence or domestic violence, we use the term interpersonal violence to highlight the intergenerational nature of violence that can occur in any existing relationship, not exclusive to intimate partners. For mothers^1^, the effects of IPV extend to their ability to parent effectively and to the quality of the parent–child relationship (Anderson & Van Ee, 2018). For the children involved, living in a home with violence, even without direct exposure, can have devastating and extensive impacts on their health, development, behavior, and capacity for healthy relationships in the future (Anderson & Van Ee, 2018; Greeson et al., 2014; McWhirter, 2011). As such, interventions are critical to support mothers and their ability to care for their children and themselves.

Despite the increased attention to IPV and some targeted interventions for mothers, the evaluation of such programs is limited. Within the evaluations, there is an almost exclusive focus on program outcomes, rather than the processes that facilitate or contribute to changes in mothering women (Micklitz et al., 2023). Although it is essential to have a deep understanding of which methods and approaches to interventions are effective, it is equally important to understand *how* these interventions influence positive changes in the participants. Highlighting the specific pathways of change within an intervention that either helps facilitate or hinders positive change can be used to support a more formalized theory of change model (Mayne, 2015), as well as allows for the optimization of direction, strategies, and focus within the intervention to meet the needs of specific populations. More generally, understanding intervention processes can support future practices and programs that lead to effective outcomes (Kazdin, 2007; Micklitz et al., 2023). In this study, using the reflections of women who participated in an existing intervention for mothers who have experienced IPV (*Connections*), we aimed to identify the processes that contributed to positive change in mothers of young children, with the goal of developing a preliminary model of perceived pathways of change. This initial model can be used as a starting point toward a theory of change, ultimately identifying relationships between the rationale, activities, and assumptions of an intervention and its expected outcomes for users—essentially, the how and why an intervention is expected to work (Ghate, 2018; Mayne, 2015).

### 1.1. Potential Pathways Supporting Change Through IPV Intervention

In addition to evaluating the impact of interventions for women who have experienced IPV, it is critical to identify pathways of change in interventions. Understanding the active processes of change will allow for the identification of core components of interventions, help illuminate barriers to change, or suggest potential leverage points that might enhance positive changes. This knowledge will help researchers, program developers, and clinicians in tailoring interventions to best suit the needs of different women in different contexts, based on the active processes. For this study, we reviewed published evaluations of existing evaluations of interventions for women who have experienced IPV to identify pathways connected to positive outcomes.

#### 1.1.1. Group and Social Support

Not only is the content of an intervention a critical component to facilitate change, but also the format in which the intervention is delivered (Craven et al., 2023; Howell et al., 2015). Given that IPV is often an isolating experience, women may appreciate group interventions which provide space to explore their shared experiences with others. Craven et al. (2023) reported that as women became aware of the support from others in an IPV group program, they developed a sense of social support and inclusion. Research indicates that participants in programs aimed at women who have experienced IPV were able to learn from each other (Craven et al., 2023; Howell et al., 2015) in areas such as becoming aware that their partner’s actions were abusive (Cluss et al., 2006), developing positive parenting strategies (Howell et al., 2015), and the awareness of external available resources (Panagioti et al., 2014). This sense of social support and the ability to respond to others’ needs were found to facilitate an improvement in the women’s self-esteem, as well as a decrease in depressive symptoms, anxiety, and PTSD (Craven et al., 2023). These changes contributed to their quality of life, which may protect against the implications of IPV (Reisenhofer et al., 2019). Cluss et al. (2006) found that *actual* support facilitates change and protects the women and even having a *perceived* sense of support may make their situations seem more manageable (Anderson & Van Ee, 2018; Greeson et al., 2014; McWhirter, 2011).

Additionally, the framework of an intervention also plays a role in the experiences and outcomes. Trabold et al. (2018) found that using a trauma-informed curriculum within an IPV intervention was essential in supporting improvement for women. For the participants, besides supporting their understanding of the impacts of trauma, knowing that the facilitators understood the effects of trauma resulted in a shared sense of safety within the group, and their willingness to share their experiences.

#### 1.1.2. Information and Awareness Leading to Cognitive Restructuring

In addition to the benefits of a group format, gaining an awareness of IPV can provide participants with tools for positive change. Trabold et al. (2018) found that for some participants, learning about the abusive nature of their partner’s behaviors was a cornerstone of their healing, allowing for new ways of thinking. For instance, learning that their partner’s behaviors were not their fault led to a shift from self-blame and shame to being able to hold their partner accountable. Recognizing that all women are vulnerable to IPV helped them reshape negative thought patterns and feelings, thus decreasing loneliness and isolation (Trabold et al., 2018). Through new knowledge, women were able to reframe their thought patterns into productive, positive behaviors and coping strategies that could support them through necessary situations such as co-parenting, contact with the perpetrator, and a shift from self-blame and responsibility to self-worth (Craven et al., 2023; Trabold et al., 2018).

#### 1.1.3. Self-Efficacy

A common pathway identified in the literature was the development of self-efficacy. Self-efficacy refers to the personal belief that one has the power to make changes in one’s life (Cluss et al., 2006), or the personal judgment one uses to execute certain actions required to deal with situations (Bandura, 2001). Self-efficacy was found to be a key pathway that enabled the participants in interventions for women who have experienced IPV to recognize their abilities and realize their capacities to make positive changes. With this realization, the women were able to identify their strengths needed to make changes and recognize their capacity to deal with difficult situations and traumatic experiences (Cluss et al., 2006). Those with a higher sense of self-efficacy tend to have the ability to persevere in the face of adversity and are more likely to take on challenges that may lead to change (Reisenhofer et al., 2019). A previous successful attempt at change lead to increases in self-efficacy (Bandura, 1977), thus supporting subsequent positive actions and behaviors. As such, a sense of self-efficacy can be the foundation of change for women and mothers in IPV interventions.

### 1.2. The Connections Intervention

*Connections: A Group Intervention for Mothers and Children Experiencing Violence in Relationships* (Breaking the Cycle, 2014) was developed in 2006 based on ongoing evaluation and the high rates of interpersonal violence reported by the participants enrolled in an early intervention and prevention program for mothers with substance use issues with their young children called Breaking the Cycle. Designed using a developmental-relational theoretical framework and delivered through a trauma-informed lens, *Connections* was developed around a two-generational strategy which considered the stability of attachment across generations. That is, addressing the mother’s needs, the child’s needs, and their relationship’s needs is required to interrupt the intergenerational transmission of relational patterns that involve interpersonal violence and child maltreatment. As such, *Connections* was designed to provide mothers with the opportunity to explore and process their past and present experiences of violence in relationships, and to explore its impact on their parenting and their children’s development through a holistic and integrated approach, with the main goals of increasing the mothers’ capacity for a positive sense of self, their relationships, and their parenting (Andrews et al., 2021, 2023). *Connections* is manualized and freely available; please contact the corresponding author to obtain a copy or visit mothercraft.ca where it will soon be available.

*Connections* is delivered within community-based projects through a weekly group format (Andrews et al., 2021). The intervention is organized into six topics that focus on the awareness of healthy versus unhealthy relationships, the impact of IPV on child development and behavior, building self-esteem and compassion for both mother and child, and the intergenerational effects of IPV (Andrews et al., 2021). Giving women the opportunity to reflect on their past and present relationships in which they experienced violence facilitates an awareness of how IPV may impact their capacity to parent effectively, hence impact their child’s development and well-being (Andrews et al., 2021).

A previous quantitative survey-based evaluation of the *Connections* intervention demonstrated a positive impact on outcomes in all core areas for the women, their children, and the mother–child relationships (Andrews et al., 2021). Using three-level random intercept models, the results indicated that the mothers significantly improved in terms of their relationship capacity, self-esteem, and self-efficacy after participating in *Connections* compared with before. Regarding parenting, mothers significantly improved in their awareness of the impact of IPV on their children, the need to build their children’s self-esteem, their role in positive parenting, and significantly decreased in parenting stress. The static group comparison indicated that improvements in the knowledge of *Connections* constructs were particularly robust (as well as knowledge of community supports and services, self-esteem, and self-efficacy). In addition to this outcome evaluation for mothers, the *Connections* facilitators reported positive change in their work, within their organization, and across their communities due to their own development of understanding and skills to address IPV (Singh et al., 2020).

### 1.3. Current Study

Despite evidence of the overall effectiveness of the *Connections* intervention in both the approach to the delivery and outcomes (Andrews et al., 2021, 2023), a knowledge gap exists in understanding the key processes that promote, support, or facilitate positive changes in the mothers within the intervention. As such, the aim of this study was to listen to mothers’ voices and reflections on the key perceived pathways of change through participation in this intervention for women who had experienced IPV. Using data from semi-structured interviews and focus groups with mothers who participated in the *Connections* intervention, we employed a phenomenological analysis to understand the mothers’ experiences and identify emerging themes, with the overarching goal of organizing changes in ways of thinking (cognition) and competencies (behavior) into perceived pathways of change. Understanding the processes leading to these shifts in cognition and competency can provide the foundation to further explore the relations between processes and intervention outcomes, leading to a theory of change.

## 2. Materials and Methods

### 2.1. Qualitative Approach

This study adopts a phenomenological approach to explore the lived experiences of mothers participating in an IPV intervention program. Using a phenomenological approach, we seek to capture the subjective meanings that participants ascribe to their experience. This method allows for an in-depth understanding of how the intervention is experienced from the participants’ perspectives and any perceived pathways of change, privileging their voices and contextual narratives.

### 2.2. Researcher Reflexivity

Our team comprises a group of women (most of the team are mothers) with varied but related educational backgrounds (e.g., psychology, social work, family and human development, and child and youth studies). The primary data analyst (MM) is a clinical psychologist and researcher with over 25 years of experience working with mothers of young children impacted by addiction and interpersonal violence; the secondary analyst and lead researcher (NA) is a community-engaged academic and has conducted research with this population for almost 10 years. As a team, our long-standing work with this population has shaped how we listen to and interpret the stories shared by participants, and each of our involvement in the study combines both professional and personal interest. We approached the interviews and analysis with deep respect for the women’s perspectives, and a commitment to centering their voices in the interpretation process. While our familiarity with the intervention, the population, and the complex issues surrounding interpersonal violence offered valuable insight, we remained mindful of potential biases and engaged in reflective practice and dialog among the co-authors throughout the research process.

### 2.3. Ethical Issues

This study was reviewed and approved by the Office of Research Ethics at York University (file # 2017-217; date of approval 4 July 2017). Prior to participation, all participants were provided with a detailed description of the study written in accessible language, including the purpose of the study, their involvement within the study, the risks and benefits, their right to withdraw at any time, and data collection and confidentiality. All identifying information was replaced with a participant code to ensure confidentiality of the participants. Participants were given the opportunity to ask questions before giving consent. In recognition of their participation and time, all participants received a $25 Visa gift card.

### 2.4. Setting

As part of a national intervention dissemination and evaluation initiative, we partnered with community-based early childhood programs across Canada. Following the systematic selection of partner sites using a readiness assessment tool (Andrews et al., 2020) service providers from partner sites attended an intensive weeklong in-person training course led by the *Connections* developers, Breaking the Cycle clinical staff, and Building Connections researchers and community partners. There were no formal requirements for service providers to join the certified facilitator training, and service providers had varied educational and professional backgrounds based on the varied nature of their organization and communities (see Andrews et al., 2020 for more details).

### 2.5. Eligibility Criteria

Mothers were invited to participate in *Connections* through conversations with community project staff, guided by a set of screening questions provided to determine whether they were a good fit for the intervention (e.g., readiness to participate and follow through) (see Andrews et al., 2021 for a full list of screening questions). In relation to IPV interventions, readiness is recognized as an important factor (Cluss et al., 2006; Jack et al., 2012), both in terms of how a woman might change as a result of the intervention, but also readiness in terms of the ability for participation in a safe and appropriate manner (Motz et al., 2019). All mothers who completed *Connections* intervention were invited to participate in an interview or focus group (no additional eligibility criteria).

### 2.6. Sampling Strategy

The mothers were invited to voluntarily take part in additional related research (participation was not connected to their access to the intervention itself). Approximately one month after *Connections* was completed, mothers who had consented and provided email addresses were contacted to inquire about their interest in participating in a focus group or an interview.

Out of the 224 mothering women who completed the *Connections* intervention, 94 provided their email addresses, giving consent for facilitators to connect them to the researchers of the study for further research participation. Overall, 43 mothers (19% of total) participated in a focus group or interview. This included 18 individual semi-structured interviews with 14 mothers (4 mothers were interviewed twice, as they participated in the intervention more than once) and 6 focus groups with 29 mothers (between 2 and 8 participants each). The remaining invitations were either declined due to scheduling conflicts or no response was given. *Connections* participants who completed focus groups/interviews were compared with mothers who completed *Connections* but did not take part in focus groups/interviews on sociodemographic variables and key quantitative variables used previously to evaluate the intervention (see Andrews et al., 2021 for full details). Participants were also compared on their level of satisfaction following the intervention, as well as on their intervention attendance. No differences were found between mothers who did or did not participate in the research.

### 2.7. Data Collection Instruments

Between April 2017 and December 2019, semi-structured interviews and focus groups were conducted via in-person, telephone, or online video calls, depending on the mother’s comfort level, geographic location, and internet availability. Interviews/focus groups took approximately one hour and were audio recorded for optimal transcription. Interviews/focus groups were facilitated by two co-investigators on the project (second and fifth author). Under their supervision and training, interviews were also supported by two research assistants who worked on the project and had a strong understanding of the *Connections* intervention and population. Mothers were asked broad, open-ended questions, and were encouraged to reflect honestly on their experiences in *Connections* (see Supplemental Materials S1 for the full list of interview questions). Questions primarily related to any change the women might have experienced in the domains of self, relationships, and parenting. The open-ended format of these questions allowed mothers to share any thoughts related to their personal experience in the intervention that they found to be important, while also leaving space for the researcher to ask for clarification or to follow up with clarifying questions of an area of interest (Alhazmi & Kaufmann, 2022; Smith & Osborn, 2003).

### 2.8. Data Processing

Audio-recorded interviews and focus groups were transcribed verbatim using an online transcription software to ensure meaning was not lost (Smith & Osborn, 2003). We used phenomenological analysis, as we were interested in exploring mothers’ subjective experiences in the *Connections* intervention from their own perspectives and words. Given prior expectations regarding the coding categories (i.e., self, relationship, and parenting) based on prior qualitative and qualitative studies, we opted for a deductive phenomenological approach. Following the steps outlined by Alhazmi and Kaufmann (2022), two research team members first identified individual meaning units and created initial coding categories on two axes. The first axis represented areas of change as a result of participating in the *Connections* intervention: interpersonal relationships, self, and parenting. The second axis represented the type of change that was described as a result of participating in the *Connections* intervention: changes in cognitions (e.g., awareness, knowledge) and changes in behaviors (e.g., competencies, functioning). After coding passages together and discussing any areas of difference, two full interviews were coded by the researchers, with a Coding Comparison Kappa indicating strong inter-rater reliability for all meaning units coded (>0.70). The two researchers then coded the remaining transcripts individually (see Supplemental Materials S2 for additional details).

### 2.9. Data Analysis and Trustworthiness

Following initial coding, another member of the research team (third author: MM) reviewed codes and subcodes, read coded meaning units within larger passages where necessary to understand women’s meaning, and worked to make sense of emerging themes (see Smith & Osborn, 2003). After developing initial themes, MM clustered and re-ordered themes with particular attention to direct and indirect *perceived pathways of change* which were identified by participants themselves. That is, themes were developed based on the women’s identification of the *Connections* content and learnings, skills that were attained, and results of these skills. MM also integrated themes into a preliminary model used to illustrate the perceived pathways of change. This thematic structure and model was then reviewed and discussed with an additional researcher (second author: NA), which included re-reading transcripts to aid interpretation and ensure meaning was not obscured, as well as to ensure that any divergent or unique views were adequately represented (Smith & Osborn, 2003).

Several techniques were used to enhance trustworthiness and credibility. The coding and thematic structure was reviewed and discussed with the director of the hosting Breaking the Cycle program, as well as a Breaking the Cycle clinician who has been delivering the Connections intervention for many years. To check for consistency and ensure validity (see Alhazmi & Kaufmann, 2022), the final thematic structure (including descriptions and representative quotes) and resulting model was also reviewed and discussed with a senior researcher (final author: DP) and an additional team member who had not been part of the data collection process (first author: KM).

## 3. Results

### 3.1. Participants

The participants (*N* = 43, 18–43 years old, *M* = 30.14 years, and *SD* = 6.31) attended *Connections* in 1 of 11 community-based projects across Canada. The majority (95%) of participants were Canadian-born and reported their ethnicity as North American (81%), Indigenous (21%), European (19%), and other ethnicities (12%) (they were able to select as many options as they identified with). Most of the participants (88%) reported the completion of high school as their highest level of education, and some with post-secondary education (67%). Most women were currently unemployed (81%), with a gross income of less than $18,000/year (63%), with common sources of income being social assistance (37%) or disability benefits (16%). Over half of the mothers were single (51%) or married/common law (23%) with 1 to 7 children (*M* = 2.16 children, *SD* = 1.38).

### 3.2. Synthesis and Interpretation

**Figure 1:**
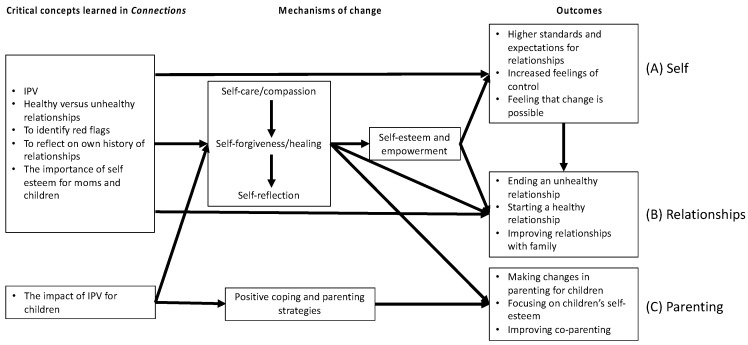
Participants’ perceptions of the pathways of change in the *Connections* intervention.

The overarching perceived pathways of change associated with this intervention for women who have experienced IPV were mapped through several processes. These included: (1) the mothers becoming aware of changes in their cognitions and behaviors following their participation in *Connections* across three domains of *self, relationships,* and *parenting* (see Table 1 for a full list); (2) the mothers recognizing the associations between their experiences in *Connections* and the changes reported in their lives; (3) integrating all addressed pathways to identify the key perceived pathways of change experienced by the mothers who participated in *Connections* (see Figure 1).

#### 3.2.1. Perceived Pathways to Change Regarding Self

The mothers were able to identify several pathways of change related to their sense of self (see Figure 1, Section A). The mothers spoke about how engaging in self-care and improving their self-compassion led to increases in their self-esteem. For instance, self-compassion emerged as an important contributor to changing thought patterns and improving self-esteem:

A lot of the way that I talk to myself has changed from the group, just stopping putting myself down and realizing that I’ve been through a lot in terms of drug abuse, alcohol abuse, and that I’m strong enough to change those negative thought patterns. And it was a really big struggle at first, but the more I practice it, the easier it becomes.

(Participant 18)

There were also several pathways identified that led to increased empowerment. One participant described how talking about IPV contributed to self-forgiveness, which allowed for self-reflection, which led to increased feelings of empowerment:

Because I think that our partners had made us feel so ashamed about what was happening that I didn’t want to talk about it. But until I was able to start talking about it, then I was able to start forgiving myself…I forgive myself, for the guilt, for the things. I didn’t do anything wrong. But I’m forgiving myself for what’s happened so I can let it go.

(Participant 15)

Another mother spoke about self-forgiveness and healing leading to empowerment. Empowerment itself also emerged as a critical theme that allowed the participants to make further changes in their lives. These changes included: having enhanced feelings of control in their lives, having the realization and ‘knowing’ that change is possible, ending relationships (*“It reinforced my belief that I needed to get out of that unhealthy relationship, and it really did make me feel like I was worth being in a healthy one.”* [Participant 24]), and having higher standards for relationships (*“It’s helped like realize some higher standards I should have had. So, I was in a different relationship at a time, and I actually ended that relationship because my newer high standards.”* [Participant 22]). Finally, the mothers spoke about how the combination of engaging in self-care along with increased feelings of empowerment allowed them to make changes for their children: *“I learned self-care…I’ve been able to apply the things to my children. So, if when I’m taking care of myself, I encourage them, to be able to teach them self-care.”* (Participant 10).

Several mothers noted that they still had *“a lot of work to do”* (Participant 12) on their self-esteem, but that these pathways of increased self-compassion, self-forgiveness, and empowerment were providing an important start:

It took a lot of work, and a lot of months but I still have self-esteem issues. I think I always will, but it gets a little bit easier, like when you’re saying positive things to yourself every day. When you’re surrounding yourself with healthy relationships, doing what’s best for you and your children and just trying to be the best you can be. Personally, that’s carried me a long way. Knowing that I have overcome so much in my past and try not to make the same mistakes as I had previously.

(Participant 19)

#### 3.2.2. Perceived Pathways to Change in Relationships

The mothers spoke about how changes to their sense of self, specifically in terms of increased self-esteem, self-compassion, and self-worth allowed them to have the strength and courage to end unhealthy relationships with a partner (see Figure 1, Section B). One participant explained it as:

I’ve attracted people that I let control me. I know now that if I want good, if I want that dream person in my head, that’s what kind of aura I need to be putting out… I have to heal myself. If I don’t heal myself, then I’ll be stuck in that position of a wounded person instead of a survivor and I quite enjoy being a survivor.

(Participant 20)

Another noted:

The group helped give me more ideas and … how to take more control because I always had the idea of how [a healthy relationship] should look like but not how to actually make it to there… because when I was in the program, I was actually in a relationship and that relationship ended shortly after the program and I kind of took more control... So I kind of made a point to be like well no like you weren’t treating me properly and this is why it can’t happen anymore kind of thing.

(Participant 2)

Improvements in self-esteem and self-worth also prompted improved relationships with other family members, including the women’s own parents.

There was also new knowledge gained that prompted changes. For instance, learning about healthy and unhealthy relationships, learning new coping skills, and moving toward increased self-worth afforded the mothers the ability to start new healthy relationships. One mother spoke about a new relationship that began after the *Connections* group:

I’ve been able to open up more and I think it’s because of the respecting of boundaries and limits and positive interactions and mutual respect…He has a healthy background and I want someone who’s healthy and that we can complement each other.

(Participant 15)

The mothers discussed how a newfound ability to identify red flags enabled them to end unhealthy relationships with a partner, as well as start new healthier relationships. For instance, one participant noted that the group helped with:

Recognizing red flags and the people around me. My children’s father. I didn’t realize that throughout the entire relationship, he was actually being very abusive. I just couldn’t see it and then when I was finally able to see it, I thought about it and I was able to leave with my children… There was finally enough, I’m like ok, I’m done dealing with all your negativity. I am done. So I grabbed my children up, we left… I’m not worthless. And I don’t deserve to be treated the way he treated me.

(Participant 4)

Another woman discussed ending a new relationship after a few dates because *“there was like some red flags that I noticed that I might not have noticed before…I was able to recognize that…I was more aware.”* (Participant 6). Finally, through reflecting on their own histories of unhealthy relationships, the mothers noted improved expectations for future relationships: *“If I start talking to someone again, I’d pay attention to more like the signs, like the red flags.”* (Participant 17).

#### 3.2.3. Perceived Pathways to Change in Parenting

The mothers spoke about how increasing their understanding of brain development led them to use more positive coping skills in their parenting role, as well as to use more strategies to build their child’s self-esteem (see Figure 1, Section C). As one participant described: *“how the prefrontal cortex isn’t nearly developed enough for them to be able to regulate their emotions. That helped me a lot to stay calm when he is having a meltdown and to let him get through… to help me stay calm, which then helped him stay calm.”* (Participant 3). The mothers also engaged in more self-care practices, which allowed them to focus on building the self-esteem of their child(ren) as well as increase their own self-forgiveness and ability to move forward in their parenting role:

I used to think it was my fault, and then I realized that it wasn’t…It did teach me that I can forgive and just let it go instead of harboring resentment…[I’m] more respectful of [my child] being a person and not just a child. Giving her more credit for what she knows and feels and what not, instead of thinking that she doesn’t understand what I’m saying-I’ve come to realize that she does.

(Participant 14)

Finally, the mothers spoke about increasing their positive parenting strategies, which led them on a pathway that included increased positive communication with their partners, encouragement and praise of the child by both parents, and increases in mindful parenting. One participant discussed how her strategies led to a closer relationship with her child: *“So, I’ve learned how to take the 5-s breath and just calmly talk to my child, and it’s made our relationship so much better. My kids communicate to me so much better than they had before, and we have a better relationship.”* (Participant 7). Another mother connected the techniques she learned with improvements in her partner’s relationship and her (and her partner’s) relationship with her child: *“[My partner and I] have gotten a lot closer and learned a lot more. We even feel closer with our baby… We’ve been using a lot of the techniques we were taught. We’ve been talking a lot more with each other.”* (Participant 18). 

#### 3.2.4. Perceived Pathways of Change

Based on the mothers’ reports of the direct and indirect changes experienced through their participation in *Connections*, we created a model to integrate and visually represent these perceived pathways (Figure 1). As shown in the model, the primary perceived pathways of change were related to shifts in a mother’s capacity for self-care and compassion, forgiveness and healing, and self-reflection. These changes also led to increased self-esteem (at least initial changes in self-esteem) and feelings of empowerment, ultimately impacting changes in their well-being, relationships, and parenting. Positive parenting strategies also supported changes in parenting and led to improvements in the parent–child relationship.

## 4. Discussion

By understanding the direct and indirect perceived pathways of change through the mothers’ own perspectives, we can optimize our direction and strategies within the *Connections* intervention. More generally, understanding intervention processes can also support practices and programs that lead to effective outcomes, as it will allow us to learn what conditions are optimal and what is needed to make positive change (Kazdin, 2007). Through phenomenological analysis, we sought to understand mothers’ experiences in the Connections intervention and identify key perceived pathways through which the mothers experienced changes as a result of their participation in *Connections*, as well as how these perceived pathways contributed to positive outcomes.

### 4.1. Maternal Changes in Cognitions and Behaviors Following Connections

Self-awareness is a crucial tool for individuals to reflect on their current states and motivate themselves to make changes for better outcomes (Carden et al., 2022). In this study, the mothers were able to describe their cognitive and behavioral changes following their participation in *Connections* across three domains of *self, relationships,* and *parenting* (see Table 1). The maternal ability to recognize these changes demonstrated their awareness of the potential harm from IPV that affected both themselves and their children. This awareness motivated mothers to prevent or avoid indicators of IPV/experiences of IPV. Moreover, the mothers could better recognize how their thoughts and behaviors differed in response to IPV-related signals that occurred within themselves, their relationships with partners, family, friends, and their children compared with before the intervention. The women observed that the personal changes they made through *Connections* led them to cultivate resilience (Mantler et al., 2024). With an awareness of the changes they experienced, researchers were able to identify common themes of perceived change that the mothers encountered through *Connections.*

### 4.2. Associations Between Connections and Maternal Changes

Following the initial development of the common themes of perceived change and the organization of intervention outcomes related to *self, relationships,* and *parenting*, the pathways of change were further mapped by associating critical concepts learned from *Connections* with the changes experienced by the mothers participating in the intervention. As noted above, Andrews et al. (2021) described that *Connections* improved mothers’ knowledge and understanding of six key *Connections* concepts, as well as enhanced their self-esteem, self-efficacy, relationship capacity, and reduced parenting stress. The mothers in the present study reported that each topic covered in *Connections* had a significant impact on the improvements they experienced, as the intervention altered their cognitions and behaviors regarding *self, relationships,* and *parenting.* The mothers’ ability to connect their learning from *Connections* to positive changes supports the effectiveness of the intervention. The mothers’ accounts highlighted the processes in which the concepts from *Connections* supported mothers exposed to IPV to address their inner turmoil and pursue resilience. The illustration of such perceived pathways is crucial in theorizing how the intervention operates and in understanding which components contribute to change (Makleff et al., 2021). The theory of perceived change derived through this study will ultimately inform the feasibility of disseminating *Connections* to prevent or intervene in cases of IPV experienced by women and their children.

### 4.3. Integration of All Perceived Pathways of Change

By integrating all the changes described by the mothers who participated in *Connections*, the perceived pathway of change for this intervention can be elucidated (see Figure 1). The key perceived pathways identified included shifts in the mothers’ capacity for self-care and compassion, forgiveness and healing, and reflection. All of these changes led to increases in self-esteem and empowerment, and ultimately effected changes in the women’s well-being, relationships, and parenting. Importantly, these perceived pathways illustrate the challenge of improving self-esteem. We learned from the women that the first step in increasing self-esteem was achieving self-compassion. They highlighted that it was the initial changes in their ability to see themselves and their experiences differently, to be kind to themselves, and to move toward forgiveness, that enabled other cognitive and behavioral changes. These findings are particularly important in light of prior work showing the importance of self-efficacy for intervention success (Cluss et al., 2006; Reisenhofer et al., 2019). According to the mothers in our sample, additional internal change is needed prior to the possibility of increasing self-efficacy. That is, self-compassion and forgiveness were needed for the mothers to feel empowered, which allowed them to feel worthy to make changes for themselves. To make positive changes, the individual needs to feel that they are worthy of the positive changes they aspire to. We also found that the internal processes related to self-compassion and self-forgiveness impacted all outcome areas, including self, relationships, and parenting. These findings highlight the importance of supporting mothers who experience IPV so they can improve their parenting and the lives and well-being of their children; support which then extends to the child(ren) involved as well.

### 4.4. Limitations and Future Directions

This study was limited in its exclusive focus on mothers who completed the *Connections* intervention. As such, the perceived pathways of change that we identified from the women’s stories are only applicable to those who were able to complete the intervention. This limitation is significant, given that our prior work indicates differences between participants who completed the *Connections* intervention (74%) compared with those who did not (26%) (Andrews et al., 2021). Specifically, women who did not complete *Connections* entered the program with increased vulnerability, including lower income, less stable housing, and lower education levels. Furthermore, the women who participated in *Connections* were themselves a specific population. As part of the recruitment process, potential participants engaged in a discussion of readiness considerations with the community facilitators of the intervention, to ensure that the intervention would be appropriate for them at that time (see Andrews et al., 2021 for more detail about this process). These intake discussions included screening questions, such as whether the women could share appropriately in a group setting and whether the women had a safe and stable relationship with their children. We recognize that the perceived pathways of change might be different for participants who were not at the same level of readiness. Nevertheless, our experience with *Connections* indicates that it is critical to consider readiness and ensure that the group program only includes participants for whom it will be safe.

This study included mothers’ reports of their own experiences in the *Connections* IPV intervention. The mothers’ self-reports provided us with an insider’s perspective of the active ingredients of the interventions based on the personal accounts of change. This qualitative method of relying on the women’s own perspectives, however, has inherent biases. As such, we use the term perceived pathway of change to allude to the fact these changes are distinctly the perceptions of any changes in these women. Future work that includes the use of objective measures along with personal accounts may enhance the validity of the findings.

Finally, the interviews and focus groups were conducted approximately 1–2 months following completion of the *Connections* intervention. In future research on the *Connections* program, it will be important to assess longer-term outcomes and determine whether the perceived pathways identified in the present study continue to support positive changes in the domains of self, relationships, and parenting over time.

## 5. Conclusions

Prior research and evaluation surrounding the *Connections* intervention has focused on outcome evaluation, wherein pre-intervention and post-intervention data from 184 mothers who completed the *Connections* intervention demonstrated significant behavioral and attitudinal shifts, particularly in the areas of mothers’ understanding of interpersonal violence, as well as their self-esteem and self-efficacy (Andrews et al., 2021). Further research, using the same sample of 43 mothers as in the current study, focused on the broader structural and implantation factors that enhanced mothers’ experiences in the *Connections* intervention (e.g., readiness considerations, group timing and structure, and other services provided; Andrews et al., 2023). In contrast to this prior work, the current study provides novel insights into the pathways through which mothers experience change in their cognitions and behaviors, as a direct result of participating in the *Connections* intervention. Based on the mothers’ own words, the pathways to change in themselves and their relationships particularly rely on changes in terms of self-compassion, self-forgiveness, and self-reflection, along with parenting changes being based on learning and practicing positive coping and parenting strategies.

These theorized perceived pathways of change emphasize the effectiveness of *Connections* by demonstrating how *Connections* is applicable to mothers experiencing IPV. This can also inform future understandings toward developing a theory of change. The findings also inform how the capacity of these mothers significantly contributes to positive outcomes in *self, relationships,* and *parenting.* Understanding these perceived pathways of change will assist clinicians and service providers in optimizing the facilitation of *Connections* within community-based projects, while supporting IPV-exposed mothers and their children in gaining access to other services within the community (Andrews et al., 2023). The findings of this study demonstrate the importance of clinicians and service providers understanding that for women to benefit from such programs and interventions, they must first be given the opportunity to develop self-compassion through reflecting on their past relationships and working toward self-forgiveness. With a foundation of self-compassion and forgiveness, women will recognize their strengths and worth and be able to benefit through the program. Women will be able to gain new knowledge and awareness of IPV, receive and provide social support with others in the group, and learn about community resources to support them and their children. This study goes beyond an evaluation of outcomes from the *Connections* program by examining the specific perceived pathways within the *Connections* program that contribute to positive change in women and in this way contributes to the growing field of intervention and program evaluation. The findings of this study provide critical guidance for a deeper understanding of how best to support mothers who are faced with the multifaced impacts of IPV in relation to themselves, their relationships, and parenting.

## Figures and Tables

**Table 1:** Participant-reported changes from intervention participation.

Domain	Type of Change	Change Identified	*N*
Self	Cognition	Increased empowerment (more confident, strong, in control, affirmed, and hopeful)	8
		Increased self-forgiveness, less blame, shame, and doubt	5
		Increased self-compassion, self-worth, and self-kindness	4
		Reflective about healing left to do	4
	Behavior	Increased self-care practices and routines (exercise, eating better, asking for support, and reflecting on group materials)	4
		Ended unhealthy relationships	4
		Increased focus on children (focus on self-esteem of child, focus on child instead of partner)	2
Relationships	Cognition	Change in expectations, what they deserve in relationships (present and future)	7
		Identifying red flags, realizing current relationship is not healthy	7
		Accepting limitations in relationships, creating boundaries	2
		Understand need to focus on themselves	2
	Behavior	Improved communication in relationships	4
		Improved boundaries	3
		Started new relationship	2
		Engaged in self-care practices	1
Parenting	Cognition	Understanding how child has been impacted by IPV	2
		Better understanding of their child (better able to understand child’s behaviors, more aware of child’s self-esteem)	2
	Behavior	Developing parenting strategies (better communication with partner; responding positively to child and with more patience; praise, encouragement, and positive feedback for child; and acknowledge and label feelings with child)	10
		Developing coping strategies for parenting (regulate own behaviors in their parenting role, self-forgiveness/less self-blame regarding parenting, and mindful parenting)	5
		Focus on self-care and self-esteem (self-care practices for parent and child, strategies to build child’s self-esteem)	4

*N* = number of participants who spoke about the identified change.

## Data Availability

The datasets presented in this article are not readily available due to privacy and ethical restrictions.

## Note

1 We use the terms ‘women’ and ‘mothers’ interchangeably because the focus of this study is women in a parenting role.

## Supplementary Materials

The following supporting information can be downloaded at: https://www.mdpi.com/article/10.3390/bs15060739/s1.
