# Supplementary material for: Perceived Pathways of Change in an Interpersonal Violence Intervention for Mothers: The Importance of Self-Compassion
Source: Behav Sci (Basel). 2025 May 27;15(6):739. doi: 10.3390/bs15060739 (PMC12189803; doi:10.3390/bs15060739)
Supplement: Supplementary file 1 [file behavsci-15-00739-s001.zip › behavsci-3582796-supplementary.pdf]

S1
Interview/Focus Group Guide

Please describe your experiences in the Connections group. What was the group like for you?

Is this group different from other groups you have attended? How so?

What do you think changed for you over the course of participating in the group? Can you think of an example of this?

Healthy relationships

a. Did your ideas/thoughts about healthy relationships change at all over your time in the group?
b. Has anything changed in your interpersonal relationships?

Self-esteem

a. Have your ideas/thoughts about your self-esteem change at all over your time in the group?
b. Do you feel like the group helped you gain strategies to support your self-esteem?
c. Have your thoughts about how you can support your child's self-esteem changed?

Parenting

a. Have your ideas/thoughts about parenting change at all over your time in the group?
b. Has anything changed in your parenting?

What was your experience like with the other women in the group?

Thinking about what changed over the course of participating (i.e., relationships, self-esteem, parenting), have things continued to change/progress after the group was over? In what ways?

Have there been additional changes in your relationships/self-esteem/parenting due to your experience in the Connections group that have occurred after the group ended?

S2
Audit Trail

Given privacy and confidentiality concerns with this highly vulnerable population of participants in an interpersonal violence intervention, recordings, transcripts, and notes will not be made publicly available. Below is a summary of the data collection, processing, and analysis process.

Raw Data

- Includes audio recordings and verbatim transcripts
  - 18 interviews
  - 6 focus groups
  - Data tracking sheet with location, Connections attendance, etc.

Data Reduction and Coding

- Initial coding using NVivo software, along 2 axes
  - Interpersonal relationships, self, parenting
  - Cognitions, behaviors
    - Cognitions include awareness, knowledge
    - Behaviors include competency, functioning
- Process
  - Training
  - Dual coding (discuss for consensus)
  - Dual coding to satisfactory reliability
  - Individual coding (SZ, CS)

Analysis Process

- Thorough review of coding (MM)
  - Codes, subcodes, meaning units
  - In narrative context
- Handwritten notes regarding initial themes; reorganization and consolidation
  - Example organization for relationships:
  - Relationship cognition (1.1-1.20)
    - Expect/deserve
    - Current relationship
    - Red flags
    - Limits/boundaries
    - Need to focus on self
  - Relationship behavior (1.1-1.17)
    - Communication
    - Boundaries
    - New healthy relationship
    - Self-care
  - Relationship pathways (1.1-1.18)
    - Self-efficacy/worth; red flags; care/compassion; coping; reflection
      - Self-esteem/compassion (worth/care) → end relationship with partner, improve family relationship

        - Red flags →end relationship; start new healthy relationship
        - Coping, learning → start healthy relationship
        - Reflection/history → expectations
- Review/reorganization in line with codes, illustrative quotes
- Review/reorganization with NA
    - Read initial transcripts in line with codes, notes, themes
    - Discussion with MM for clarification, areas of disagreement
    - Initial thematic structure (MM)
- Peer feedback and amendments
    - Online meeting with ML, GD, DP, MM, NA; review documents, thematic structure; illustrative quotes
    - Modifications (MM)
    - Online meeting (same attendees) to review modifications, any additional changes

Final Report

- Begin writing report (results section of manuscript)
    - Initial drafts NA, discussions/edits from MM
    - Review of report, in line with coding, thematic structure by KM
